# Isotyping and Semi-Quantitation of Monkey Anti-Drug Antibodies by Immunocapture Liquid Chromatography-Mass Spectrometry

**DOI:** 10.1208/s12248-020-00538-w

**Published:** 2021-01-06

**Authors:** Xiaoxiao Huang, Xiaobin Xu, Michael A. Partridge, Jihua Chen, Ellen Koehler-Stec, Giane Sumner, Haibo Qiu, Albert Torri, Ning Li

**Affiliations:** 1 Analytical Chemistry, Regeneron Pharmaceuticals Inc., Tarrytown, New York 10591 USA; 2 Bioanalytical Sciences, Regeneron Pharmaceuticals Inc., Tarrytown, New York 10591 USA

**Keywords:** ADA, immunogenicity, isotyping, LC-MS, monoclonal antibody

## Abstract

There is an urgent demand to develop new technologies to characterize immunogenicity to biotherapeutics. Here, we developed an immunocapture LC-MS assay to isotype and semi-quantify monkey anti-drug antibodies (ADAs) to fully human monoclonal antibody (mAb) drugs. ADAs were isolated from serum samples using an immunocapture step with the Fab of the full-length mAb cross-linked to magnetic beads to minimize matrix interference. A positive monoclonal antibody control against the human immunoglobulin kappa light chain was used as a calibration standard for ADA quantitation. The final LC-MS method contains 17 multiple reaction monitoring (MRM) transitions and an optimized 15-min LC method. The results suggested that IgG1 was the most abundant isotype in ADA-positive samples. IgG2 and IgG4 were identified at lower levels, whereas IgG3 and IgA levels were only observed at very minor levels. In addition, levels of total ADA measured by the LC-MS assay were comparable to results obtained using a traditional ligand binding assay (LBA). The LC-MS ADA assay enabled rapid immunogenicity assessment with additional isotype information that LBAs cannot provide.

**Supplementary Information:**

The online version contains supplementary material available at 10.1208/s12248-020-00538-w.

## INTRODUCTION

Biotherapeutics, such as monoclonal antibodies (mAbs), have been rapidly growing in the pharmaceutical market. However, these therapeutics have the potential to elicit an unwanted immune response that can result in the production of anti-drug antibodies (ADAs) (1–3). The binding of ADAs to a biological product can decrease the half-life of the product and/or have neutralizing activity. ADAs can therefore potentially reduce drug efficacy and pose concerns for patient safety.

A study of 121 US Food and Drug Administration (FDA)–approved biological products revealed that 89% of products showed ADA incidence, with almost half reporting some impact on efficacy, although only 26% reported impacts on PK (4). The generation of ADAs can be caused by product- or process-related factors such as the molecular structure of the biotherapeutic, product purity, post-translational modifications, dose, route, or frequency of administration (5,6). ADAs can also be induced by patient-related factors including genetic profile, immune status, or disease status (7).

Although immunogenicity in animal models is not predictive of immunogenicity in humans, nonclinical immunogenicity studies are still informative for the evaluation of the impact of ADAs on pharmacokinetics and drug safety (8). Humans have five immunoglobulin isotypes including IgM, IgG, IgA, IgE, and IgD. Among them, IgM responds to the initial exposure of antigens, normally appearing within 7 days. IgG is the most abundant antibody and responsible for the long-term immune response to antigens. IgG has 4 subclasses including IgG1, IgG2, IgG3, and IgG4. IgA is mostly secreted into mucus, tears, and saliva. IgE is the least abundant isotype but it plays critical roles in allergic reactions and antiparasitic activity. IgD is mainly expressed on the surface of immature B cells (9). Similar to humans, monkeys also have five immunoglobulin isotypes, which include IgM, IgG, IgA, IgE, and IgD (10).

Detection and analysis of ADA formation are important for understanding immunogenicity and patient immune response in biological therapeutics development (11). Thus, the impact of ADA on product safety and efficacy of biological therapeutics should be evaluated during development. Additional immunogenicity assessments, such as isotyping, epitope mapping, and cross-reactivity may also be required by regulators. Commonly used methods for ADA detection are ligand binding assays (LBAs) using an electrochemiluminescence (ECL)-based bridging format (7,12). In these assays, ADA forms a bridge between drugs labeled with two different tags or haptens, and these immune complexes are captured on a plate resulting in a signal in the assay. Although LBA provides high throughput, high sensitivity, and good specificity, interference from drugs, targets, and other serum components might cause false-positive or false-negative results. In addition, these methods may not be able to detect low abundant ADAs. Importantly, singleplex ligand binding assays are not capable of ADA isotyping or quantitation.

Surface plasmon resonance (SPR) is another widely used method to measure drug and ADA interaction in “real-time” (12), where serum samples flow through a drug-immobilized sensor chip and the changes in the refractive index are detected as a result of drug-ADA interactions. SPR can detect low-affinity antibodies for early immune response and enable isotyping and kinetics analysis. However, SPR has low throughput and low sensitivity.

With the rapid development of new instrumentation and methods, liquid chromatography-mass spectrometry (LC-MS) has emerged as a critical tool for the characterization of biologicals (13–15). Many efforts have been made to adopt the LC-MS-based approach as a supplementary tool for ADA analysis (16). One study used a bead-based immunoprecipitation method followed by LC-MS to assess ADA to human growth hormone analog (hGHA) (17) in order to overcome drug interference issues that are associated with conventional ADA assays. In this study, an excess amount of drug was used to saturate ADA binding sites. The drug-ADA complex was then captured by protein G conjugated magnetic beads, followed by elution and digestion with cyanogen bromide (CNBr). The CNBr-released drug peptides were quantified by LC-MS, enabling indirect quantitation of total ADAs.

In another study, bead extraction and acid dissociation (BEAD) were paired with LC-MS to simultaneously quantify the residual mAb drug, endogenous human IgG, and the neutralizing antibody (NAb) (18). This approach efficiently removed high concentration drug and serum components, which can interfere in different analytical methods, and demonstrated the advantage of LC-MS in selectivity and multiplexing, providing a fast and direct approach to characterize multiple components for an immunogenicity assessment.

More recently, an immunocapture LC-MS method was developed for simultaneously isotyping and semi-quantitating pre-existing ADAs in human plasma (19). In this method, two strategies were used for ADA immunocapture. The first one used biotinylated drugs as the capture reagent to pull down ADAs. The second one took advantage of biotinylated anti-drug mAb to form mAb-drug-ADA complexes. The captured ADAs were immobilized on streptavidin magnetic beads so that they could be separated from human plasma. The eluted ADAs were digested and followed by LC-MS detection of specific universal peptides for each ADA isotype, demonstrating the ability of the immunocapture LC-MS method to detect pre-existing ADA in human plasma.

Subsequently, a similar immunocapture LC-MS method was developed for ADA identification in cynomolgus monkey plasma (20). Roos and his colleagues used a biotinylated mouse anti-drug mAb to capture ADA-drug complexes and isolated them by streptavidin magnetic beads. A common peptide for monkey IgG1–4 subclasses was used to detect the isolated ADAs. Shortly after, the same group developed a sequential immunoaffinity LC-MS assay to quantify therapeutic proteins in monkey plasma (21). For the first immunocapture step, a biotinylated mouse anti-drug antibody was used to capture the therapeutic protein in plasma. Following tryptic digestion, a unique peptide from the therapeutic protein was captured by the second immunocapture step using a mouse anti-peptide antibody, followed by LC-MS analysis. The sequential immunocapture steps can almost completely remove the matrix interference, providing a highly sensitive method to detect low abundant ADA in plasma samples. In addition, the use of an automated magnetic bead handler enables high throughput analysis.

Another study used blue native polyacrylamide gel electrophoresis (BN-PAGE) to separate human antibody-specific immune complexes from unbound therapeutic antibodies and endogenous antibodies in monkey serum samples. The target fractions were cut from the gel and following in-gel digestion and LC-MS enabled a semi-quantitative analysis of humanized antibodies that were captured by ADAs (22).

In this study, we developed an immunocapture LC-MS method for ADA isotyping and semi-quantitation in serum samples from cynomolgus monkeys administered fully human mAb therapeutics. An ADA-positive control (ADA-PC) was used for assay development and to establish a calibration curve for semi-quantitation. The immunocapture procedure was optimized to maximize the recovery rate and minimize the nonspecific interaction with serum components. The captured ADAs were digested and analyzed by multiple reaction monitoring (MRM)–based LC-MS method. The method was successfully applied to isotype and semi-quantify ADAs in monkey serum samples from single-dose and multiple-dose nonclinical studies. The total ADA responses measured by the LC-MS assay were consistent with responses obtained with a conventional LBA. In this study, the ADA-PC spiked-in monkey sera used to generate the calibration curve went through the same immunocapture LC-MS procedure as the nonclinical samples, which we believe is the first reported effort of LC-MS-based ADA semi-quantitation. Comparing with previous efforts, the immunocapture optimization significantly increased assay sensitivity and reliability, allowing the LC-MS-based assay to obtain similar detection limits with traditional LBA.

## EXPERIMENTAL SECTION

### Reagents and Chemicals

The therapeutic drug candidate molecules, REGN10446P2 and REGN7798N, referred to hereafter as REGN-P and REGN-N, respectively, are fully human IgG4 mAbs. The therapeutic drug candidate molecules, REGN316P and REGN3592P3, referred to hereafter as REGN-6P and REGN-2P, respectively, are fully human IgG1 mAbs. REGN-ADA-PC, referred to hereafter as ADA-PC, is a monkey IgG1 mAb against the human kappa light chain. These antibodies were generated by Regeneron Pharmaceuticals, Inc. (Tarrytown, NY). Purified monkey IgA, IgG, and IgM were purchased from Life Diagnostics, Inc. (West Chester, PA). Cynomolgus monkey sera were obtained from BioIVT (Westbury, NY). Bovine serum albumin (BSA) was purchased from Millipore-Sigma (St. Louis, MO). HEPES buffered saline-EDTA polysorbate (HBS-EP) buffer was purchased from GE Healthcare (Pittsburgh, PA). Tween-20 was purchased from Bio-Rad (Hercules, CA). All other chemicals were purchased from Sigma Aldrich (St. Louis, MO) unless specified.

### Nonclinical Sample Information, Ligand Binding Drug Concentration, and ADA Assays

REGN-P serum samples were obtained from a multiple-dose toxicology study in cynomolgus monkeys. Animals received 1 mg/kg REGN-P weekly (QW) via intravenous (IV) injection. Serum samples were collected at pre-dose, post-dose at 5 min, 1 day, and 3 days within the first dosing interval, post-dose at 7, 15, 29, 43, 57, 71, 85, 106, 120, 134, 148, and 162 days. REGN-N serum samples were obtained from a single-dose pharmacokinetic (PK) study in cynomolgus monkeys. Animals received a single IV injection of 1 mg/kg, 5 mg/kg, or 15 mg/kg REGN-N. Serum samples were collected at pre-dose and post-dose at 5 min, 1, 4, 8, 12, 24, 48, 72, 96, 120, 144, 168, 216, 240, 264, 288, 312, 336, 384, 432, 480, 528, 576, 624, 672, 720, 768, 816, 864, 912, 960, 1008, 1056, 1104, 1152, 1200, 1272, and 1344 h following the end of infusion. Serum samples were stored at − 80 °C prior to analysis.

REGN-P and REGN-N serum concentrations in serum were measured using a target capture enzyme-linked immunosorbent assay (ELISA). Microtiter plates were coated with the respective drug targets and the therapeutic captured on the plate was detected using a biotinylated mouse anti-human IgG4 monoclonal antibody, followed by Neutravidin conjugated with horseradish peroxidase. A luminol-based substrate, specific for peroxidase, was added to generate a signal intensity that is proportional to REGN-P and REGN-N concentration.

Anti-REGN-P antibodies in monkey serum samples were detected using an ECL-based bridging immunoassay. Serum samples were diluted 1:10 with 300 mM acetic acid to dissociate antibody-drug complexes and were then diluted 1:3 with a 150-mM Tris solution containing biotinylated and ruthenium-labeled REGN-P. ADAs in the serum samples form immune complexes with the labeled mAbs. The samples were added to a streptavidin-coated MSD microplate (Meso Scale Diagnostics, Rockville, MD). The bound complexes were detected by an electrochemiluminescent signal using an MSD plate reader (Meso Scale Discovery, Rockville, MD).

### Selection of Quantifying Peptides and Confirming Peptides for LC-MS/MS Assay

Due to high homology shared between monkey and human IgGs, sequence alignment was performed to determine the unique peptides of each cynomolgus monkey IgG subclass (23,24). Tryptic peptides derived from purified cynomolgus monkey IgG, IgA, and IgM were analyzed by LC-MS/MS using data-dependent acquisition (DDA) method on an Acquity I-Class UPLC system (Waters, Milford, MA) coupled to a Q Exactive Plus mass spectrometer (Thermo Fisher Scientific, San Jose, CA). Unique peptides for each isotype were selected to generate an MRM method. The most abundant b and y ions after the collision for each unique peptide were used to develop the MRM transitions, with the collision energy optimized for each transition. Tryptic peptides from purified monkey isotypes were analyzed using the MRM method on a 6495 triple quadrupole mass spectrometer (Agilent Technologies, Santa Clara, CA). The unique peptide for each ADA isotype with the strongest signal was chosen as the quantifying peptide (Table I) and any additional peptides were used as confirming peptides to certify the presence of ADA isotypes (Table S1). The top 2 transitions for each quantifying peptide were used for quantitation.

**Table I:** List of Cynomolgus Monkey ADA Isotypes, Quantifying Peptides, MRM Transitions and Optimized Collision Energies Used in the Immunocapture-LC-MS Assay

Isotype	Peptide	Precursor	Product ion	Collision energy	Note
IgG1	GPSVFPLAPSSR	607.8300	874.4781	16.8	Unique for IgG1; quantifying peptide
			727.4097	16.8	
IgG2	GPSVFPLASCSR	639.3190	937.4560	23.8	Unique for IgG2; quantifying peptide
			790.3876	23.8	
IgG3	GPSVFPLVSCSR	653.3346	965.4873	27.3	Unique for IgG3; quantifying peptide
			818.4189	27.3	
IgG4	GPSVFPLASSSR	602.8197	864.4574	22.7	Unique for IgG4; quantifying peptide
			717.3890	22.7	
IgM	QIEVSWLR	515.7876	789.4254	17.0	Unique for IgM; quantifying peptide
			561.3144	17.0	
IgA	DPSGATFTWTPSSGK	769.8597	863.4258	30.9	Unique for IgA; quantifying peptide
			475.2511	30.9	

### Preparation of ADA-PC Standards

ADA-PC standards in pooled monkey serum were prepared by performing a 3-fold serial dilution using pooled naïve monkey serum; final concentrations were 10 μg/mL, 3.3 μg/mL, 1.1 μg/mL, 370 ng/mL, 123 ng/mL, 41 ng/mL, 14 ng/mL, and 1.5 ng/mL. The standards were incubated at room temperature for 30 min before immunocapture.

### Preparation of Samples for Assay Drug Tolerance Assessment

The drug tolerance samples were prepared by diluting REGN-P or REGN-N into pooled naïve monkey serum containing 500 ng/mL ADA-PC in a 2-fold serial dilution, resulting in a final concentration of drug ranging from 0.38 ng/mL to 10 μg/mL.

### Immunocapture with Biotinylated Drug

Equal volumes of the individual monkey serum sample and 600 mM acetic acid were combined and incubated at room temperature for 1 h. The acidified serum samples were then diluted with HBS-EP buffer + 0.2% Tween-20. Biotinylated REGN-P or REGN-N (produced in-house, 2 μg/sample) was added to each serum sample (final volume of 500 μL) and incubated for 1 h at room temperature to form a complex with ADA-PC or ADAs. Ten (10) microliter of Dynabeads MyOne™ Streptavidin T1 magnetic beads (Thermo Fisher Scientific, Waltham, MA) were incubated with 3% BSA in HBS-EP + 0.2% Tween-20 buffer for 30 min before being added to the serum samples and subsequently incubated at room temperature for 15 min. The samples were then washed twice with 3% BSA in HBS-EP + 0.2% Tween-20 buffer followed by 2 washes with HBS-EP + 0.2% Tween-20 buffer and elution with 0.1% formic acid (FA) and 50% acetonitrile.

### Immunocapture with Cross-linked Drug

REGN-P and REGN-N were cross-linked to streptavidin beads using Dynabeads™ Antibody Coupling Kit (Thermo Fisher Scientific, Waltham, MA) according to the manufacturer’s protocol to a final concentration of 50 ng/mL. The beads with cross-linked MABs (40 μL) for each sample were pre-blocked by incubating with 3% BSA in HBS-EP + 0.2% Tween-20 buffer for 30 min. Acidified serum samples were diluted using HBS-EP buffer with 0.2% Tween-20 (total volume of 500 μL) and added to the pre-blocked cross-linked MABs/bead solution. The mixture was incubated at room temperature for 1 h to form MAB-ADA or MAB-ADA-PC complexes. The complexes on the magnetic bead were washed twice with 3% BSA in HBS-EP + 0.2% Tween-20 buffer followed by two washes of HBS-EP + 0.2% Tween-20 buffer and elution with 0.1% formic acid (FA) and 50% acetonitrile.

### Trypsin Digestion

The eluted ADA-PC or ADAs were dried down in a SpeedVac concentrator and then resuspended in 20 μL of 100 mM Tris-HCl pH 7.4 containing 8 M urea and 10 mM of tris(2-carboxyethyl) phosphine (TCEP). The samples were incubated at 37 °C for 30 min. Trypsin (Promega, Sunnyvale, CA) was used at an enzyme: substrate ratio of 1:10, and 2.5 mM iodoacetamide was added for alkylation. The samples were incubated in the dark at 37 °C for 3 h. The digestion was terminated by the addition of 1 μL of 20% formic acid. Twenty (20) microliter aliquots of the subsequent peptides were used for analysis.

### Mass Spectrometry and Data Analysis

LC-MS/MS analyses were performed using an Acquity UPLC CSH C18 1.7 μm, 2.1 mm × 50 mm column (Waters, Milford, MA) at 40 °C on a 1290 Infinity II LC System (Agilent, Santa Clara, CA) coupled with an Agilent 6495 triple quadrupole mass spectrometer. Mobile phase A consisted of 0.1% formic acid (FA) in water, and mobile phase B was composed of 0.1% FA in acetonitrile. Tryptic peptides were separated at a flow rate of 0.4 mL/min with a linear gradient from 5% mobile phase B to 25% mobile phase B over 8 min. The column was then washed at 90% mobile phase B for 1 min and re-equilibrated to 5% mobile phase B for 2 min with a total analysis time of 15 min. The MRM mass spectrometer settings were as follows: polarity was positive mode, MS1 and MS2 resolution was wide unit, dwell time was 50 ms, and 12 quantifying and 16 confirming MRM transitions were monitored at various collision energy as described in Table I and Table S1, respectively. The MRM-MS data was analyzed using Skyline (v4.1, MacCoss Lab, the University of Washington in Seattle, WA). Total peak areas from two fragment ions for each quantifying peptide were used for quantitation.

## RESULTS

### Immunocapture LC-MRM-MS Workflow for ADA Isotyping and Semi-Quantitation

**Fig. 1:**
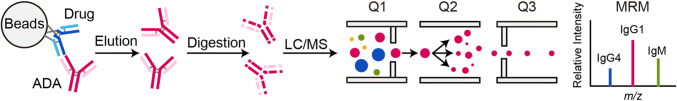
Experimental workflow. Beads with cross-linked mAb drugs are added to the serum samples to form a complex with ADAs. Bound ADAs are then washed, eluted, digested, and analyzed using an LC-MRM-MS method for isotyping and quantitation

Since singleplex bridging immunogenicity assays are not capable of determining ADA isotypes, we wanted to use the LC-MS platform to simultaneously detect, isotype, and quantitate ADA responses in monkeys administered different mAb therapeutics. MRM, also known as selected reaction monitoring (SRM), is a powerful and reliable technology to quantify low abundant proteins in complex matrices (25,26). Here, we used human mAb drugs as the capture reagents to isolate ADA from monkey serum samples. The bound drug-ADA complexes were washed to eliminate contamination from serum proteins, specifically endogenous immunoglobulins. The isolated ADAs were then digested with trypsin to generate proteolytic peptides and analyzed in the triple quadrupole mass spectrometer using an MRM method (Fig. 1). A calibration curve generated by the ADA-PC was used to quantitate the amount of each isotype (Fig. 4).

### Selection of Surrogate Peptides

In this MRM method, predefined transitions (precursor and fragment ion pair) were used to monitor and quantify specific proteins. A unique surrogate peptide corresponding to a specific immunoglobulin isotype was selected to generate this MS method. Similar to humans, monkeys have five heavy chain isotypes: IgG, IgA, IgM, IgE, and IgD; and two light chain isotypes: kappa and lambda. Monkeys also have four IgG subclasses, IgG1, IgG2, IgG3, and IgG4. Since IgD is mainly present in plasma membranes and rarely found in serum, it was not included in this assay. In addition, IgE contributes to less than < 0.003% of total immunoglobulin in serum and was therefore excluded from this assay as well.

Several criteria were used for the selection of surrogate peptides. First, the surrogate peptides of each isotype/subclass must be within the constant regions of the antibody. Second, the peptides must be unique to each isotype/subclass and unique to monkey species. Third, they should generate strong MS signals. And finally, the peptides should not bear certain post-translational modifications, chemically induced modifications, and cleavage sites. For example, peptides with glycosylation sites or those containing methionine that is prone to oxidation were avoided. In addition, peptides with two neighboring basic amino acids (RR or KK), lysine-proline (KP), or arginine-proline (RP) were avoided to minimize inconsistent trypsin digestion. Peptides that met these criteria were measured by MS. The peptide with the most intense signal for precursor and fragment ions was chosen as the quantifying peptide for each isotype. An example of the MRM transition development was shown in Fig. S1. One or two additional peptides, other than the quantifying peptide if available, were used as confirming peptides to further certify the presence of ADA isotypes. The complete list of surrogate peptides and transitions for quantifying and confirming are listed in Table I and Table S1, respectively.

### Cross-linking Drug to Streptavidin Magnetic Beads Significantly Increases ADA Immunocapture Recovery Rate Compared to Binding Biotinylated Drug to Streptavidin Magnetic Beads

Since the serum is a highly complex matrix, immunocapture enrichment was required to achieve the sensitivity necessary to distinguish potentially low abundant ADAs from other endogenous immunoglobulins using a standard LC-MS method. We compared two immunocapture approaches: biotinylating mAb drug with streptavidin-coated magnetic beads and directly cross-linking mAb drug to magnetic beads. A monkey anti-human kappa light chain IgG1 monoclonal antibody, ADA-PC, was used as a positive control to evaluate the recovery rate. To accurately assess the immunocapture recovery rate, mouse serum, instead of monkey serum, was used as the matrix to eliminate the interference from nonspecific binding of endogenous monkey immunoglobulins to ADA-PC.

**Fig. 2:**
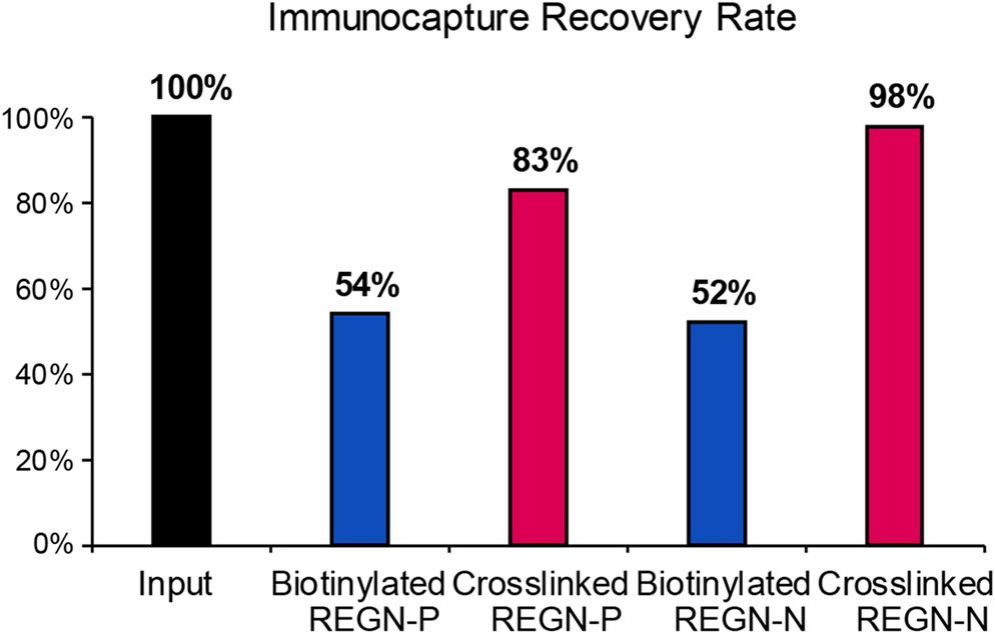
Cross-linking drug to magnetic beads significantly increases ADA immunocapture recovery rate. Two IgG4 mAb drugs REGN-P and REGN-N were used as capture reagents. Biotinylated drugs coupled to streptavidin-coated beads and drugs directly cross-linked to beads were used to immunocapture ADAs in mouse serum. The input is the same amount of ADA-PC that did not go through immunocapture procedures but went through the same digestion procedures as the samples

First, we tested the biotinylated drug’s ability to capture ADAs in sera using a method that has also been reported by others (19). Like ECL or ELISA-based ligand binding assays, immobilized streptavidin beads were used to isolate the drug-ADA complexes. Two biotinylated drugs, REGN-P and REGN-N, were used to immunocapture ADA-PC from serum samples. Compared to the input, which represents the amount of ADA-PC that was not subjected to immunocapture procedures but went through the same digestion and LC-MS procedures as samples, about 50% of the ADA-PC was detected after immunocapture for both REGN-P and REGN-N (Fig. 2). Second, we cross-linked each drug directly to magnetic beads before incubating them with serum samples containing ADA-PC. The immunocapture recovery rate was improved significantly, with REGN-P achieving 89% and REGN-N achieving 98% compared to the input (Fig. 2). Our results suggested that the direct cross-linking approach significantly improved the immunocapture recovery rate of ADA from mouse serum. Thus, the cross-linking approach was used in the final workflow.

### Using Fab-Only Drug as the Capture Reagent Significantly Reduced Nonspecific Binding to the Fc Region of IgG4 Drugs

**Fig. 3:**
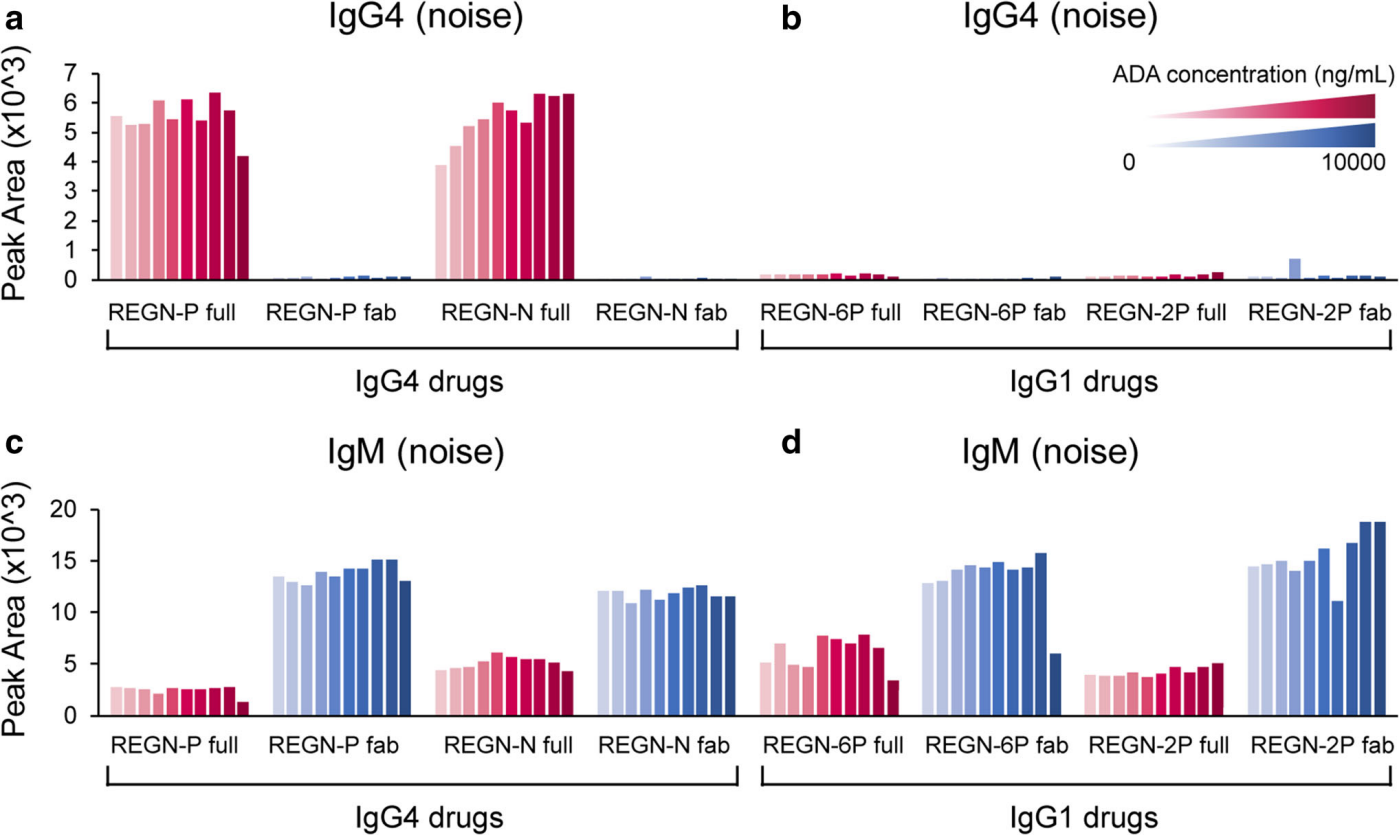
Fab-only drugs eliminate nonspecific interactions with serum components to the Fc region of IgG4 drugs. (**a**, **b**) Two IgG4 full-length mAb drugs and Fab-only drugs, and two IgG1 full-length mAb drugs and Fab-only drugs were used as capture reagents. In contrast to IgG4 drugs, IgG1 drugs do not have high IgG4 noise. IgG4 Fab-only drugs have significantly lower IgG4-mediated noise comparing to IgG4 full drugs. (**c**, **d**) For both IgG4 and IgG1 drugs, Fab-only drugs cause higher IgM-mediated background noise

Human IgG4 antibodies are known to interact with other immunoglobulins via their Fc domain and can generate substantial background noise in immunoassays (27–29). Monkey IgG4 in serum samples may also bind via its Fc to the IgG4 drug cross-linked to magnetic beads in the immunocapture step, generating background in the assay. To test this, we compared full-length REGN-P and REGN-N to their corresponding Fab versions for the ADA immunocapture. Notably, high IgG4 background noise levels were detected when using full-length mAbs in the immunocapture step. This was substantially reduced when using Fab fragments to isolate ADAs (Fig. 3a). Two IgG1 drugs, REGN-6P and REGN-2P, were also tested with the full-length and Fab versions to determine if other isotypes could generate similar background noise. As expected, the two IgG1 drugs did not generate high IgG4 background noises (Fig. 3b).

In contrast, IgM matrix interference was observed for both IgG1 and IgG4 drugs (Fig. 3c & d), when both full-length mAb and Fab were used in the immunocapture step. Interestingly, there were greater levels of IgM background noise when the Fab was used for immunocapture. Because of the significant reduction of nonspecific interactions, Fab-only drugs cross-linked to magnetic beads were used as the immunocapture reagents in the final workflow.

### Determination of LLOQ and ULOQ of the Assay Using a Standard Calibration Curve

**Fig. 4:**
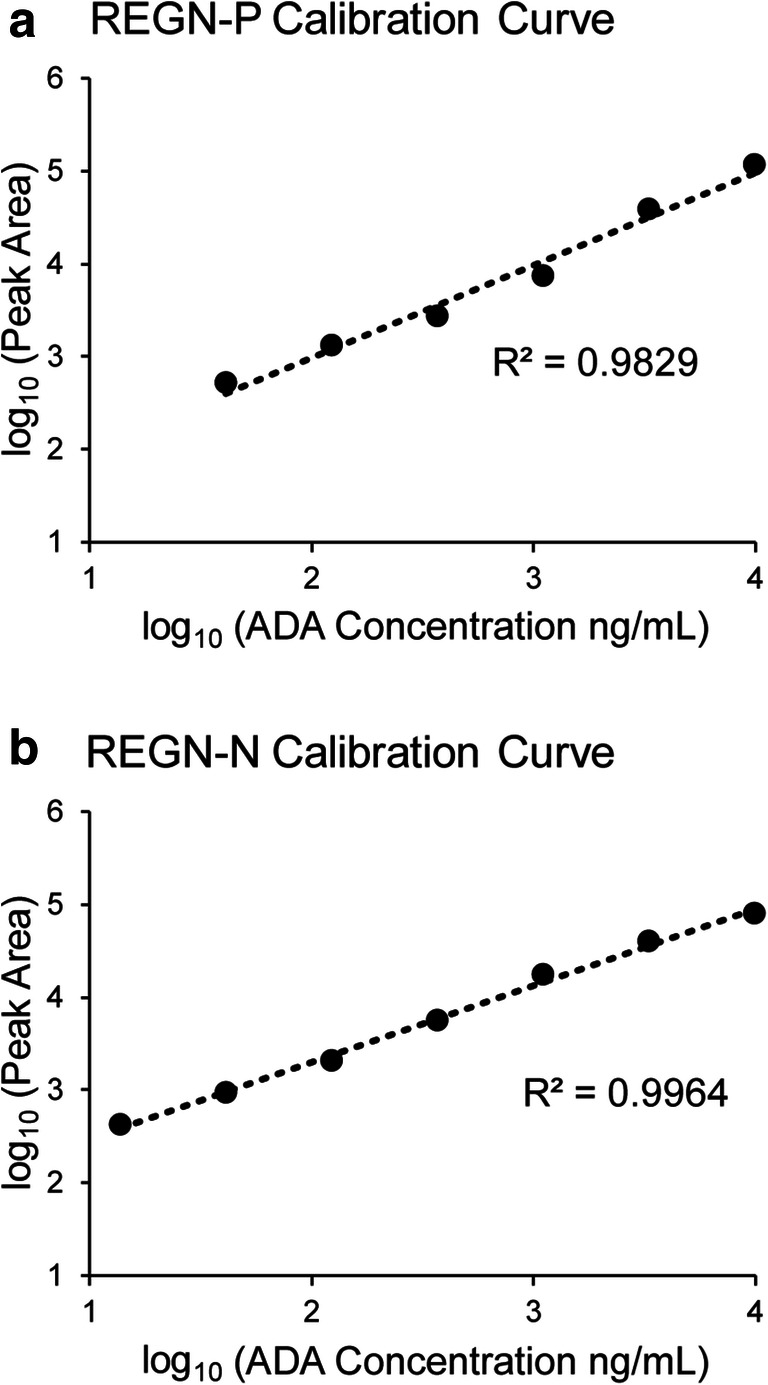
ADA calibration curves of REGN-P and REGN-N. Calibration curve of REGN-P (**a**) and REGN-N (**b**). The *x*-axis represents the log scale of ADA concentration while the *y*-axis represents the log scale of peak area of IgG1 quantifying peptide, GPSVFPLAPSSR, quantified using the MRM method. The lowest point in (**a**) corresponds to 41 ng/mL of ADAs. The lowest point in (**b**) corresponds to 14 ng/mL of ADAs

Standard calibration curves used for ADA quantitation were developed for both REGN-P and REGN-N drugs. ADA-PC was serially diluted with pooled naïve monkey serum from 10 μg/mL to 1.5 ng/mL, using a 3-fold dilution factor. The positive control samples were processed with the optimized immunocapture method procedure with cross-linked REGN-P Fab and cross-linked REGN-N Fab as the capture reagents. The isolated ADA controls were then analyzed by the 15-min LC-MS method in triplicate. MRM transitions of GPSVFPLAPSSR peptide from IgG1 were used for quantitation (Table I). The peak area corresponding to each ADA-PC concentration was used to plot the calibration curve. The REGN-P calibration curve showed a linear range from 41 ng/mL to 10 μg/mL (Fig. 4a) and the REGN-N calibration curve showed a linear range from 14 ng/mL to 10 μg/mL (Fig. 4b). For REGN-P, the limit of detection (LOD) was determined to be 1.5 ng/mL using a single-to-noise (S/N) ratio of 3 and the lower limit of quantitation (LLOQ) was determined to be 41 ng/mL using an S/N ratio of 10, as well as in the linear range of the calibration curve. For REGN-N, the limit of detection (LOD) was determined to be 1.5 ng/mL using a single-to-noise (S/N) ratio of 3 and the lower limit of quantitation (LLOQ) was determined to be 14 ng/mL using an S/N ratio of 10, as well as in the linear range of the calibration curve. The upper limit of quantitation (ULOQ) for both REGN-P and REGN-N were determined to be 10 μg/mL as the upper limit of the linear range of standard calibration curves.

### Determination of Drug Tolerance Limit

The presence of therapeutic drugs in nonclinical monkey serum samples may compete with immunocapture reagents for binding to ADAs, also known as drug interference. To assess the assay tolerance to REGN-P and REGN-N drugs present in serum samples, pooled monkey serum samples containing 500 ng/mL of ADA-PC were tested in the presence of different concentrations of REGN-P or REGN-N (ranging from 0.38 ng/mL to 10 μg/mL) to determine what concentrations of drug impacts on ADA quantitation and ADA detection.

**Fig. 5:**
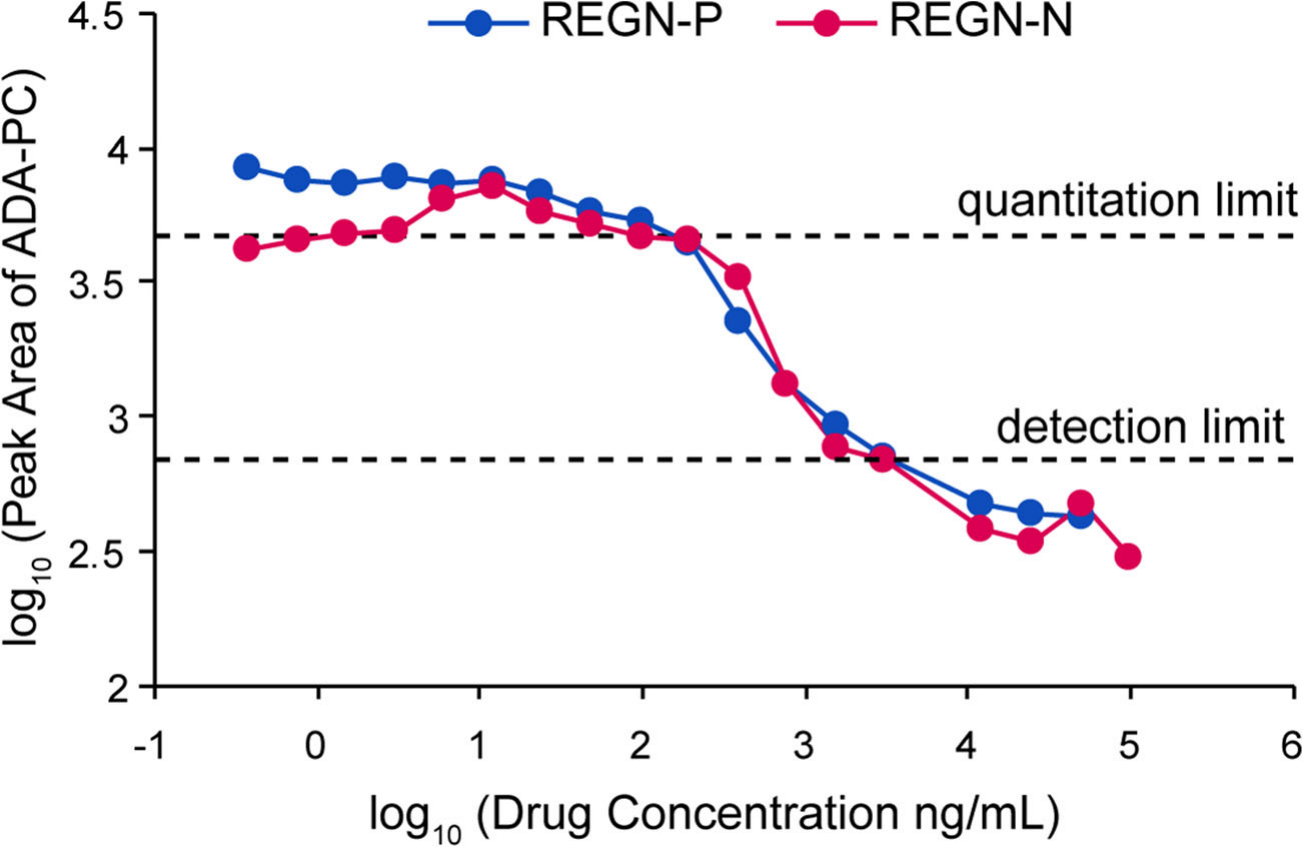
Determination of drug tolerance limit. REGN-P or REGN-N was diluted into pooled naïve monkey serum containing 500 ng/mL of ADA-PC in a 2-fold serial dilution, resulting in a final concentration of drug ranging from 0.38 ng/mL to 10 μg/mL. ADA levels were measured to assess the impact of the drug concentration on ADA quantitation and detection limits

The quantitation of ADA-PC was not impacted by REGN-P or REGN-N until the drug concentration reached 195 ng/mL (Fig. 5). When the drug concentration was higher than 195 ng/mL, the concentration of ADA-PC detected in the assay started to decrease, indicating the competing effect of the drug in the sera for ADA binding.

Ligand binding ADA assays are non-quantitative and ADA concentrations in nonclinical studies are reported as either negative or positive with a titer (dilution) value which provides a relative quantitation of the antibody responses. In the drug tolerance experiments, ADA-PC can still be detected even at REGN-P or REGN-N concentration of 3.1 μg/mL, with the ADA-PC signal 3-fold greater than assay background at this drug concentration.

### Application of Optimized Method to Nonclinical Monkey Serum Samples for ADA Isotyping and Semi-Quantitation

**Fig. 6:**
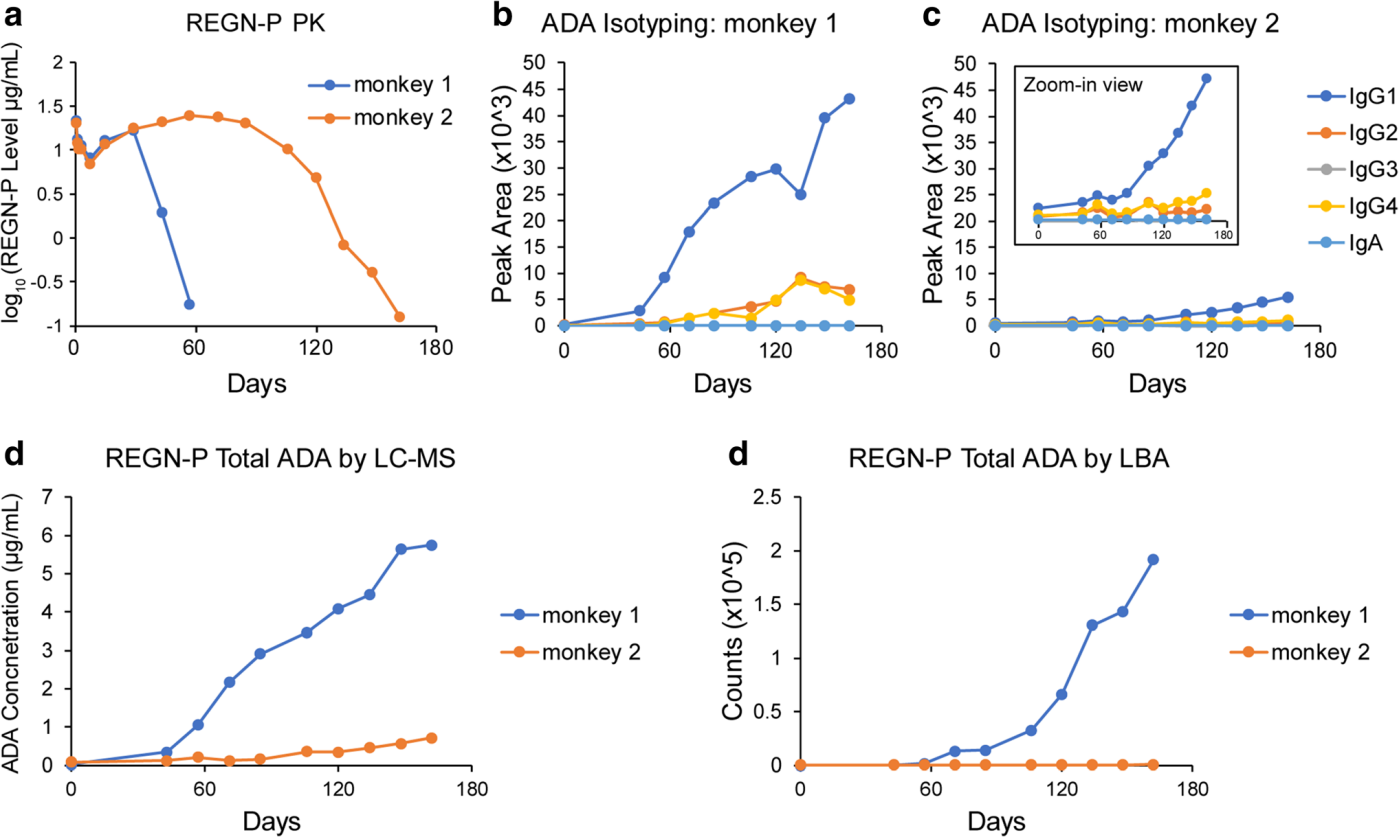
REGN-P monkey serum sample ADA isotyping and quantitation. **a** PK results of two monkeys with weekly dose of 1 mg/kg REGN-P for 23 weeks. **b** ADA isotyping results of monkey 1 using the immunocapture LC-MS method. **c** ADA isotyping results of monkey 2 using the immunocapture LC-MS method. **d** Total ADAs were quantified using the calibration curve in Fig. 4a to convert the peak areas to ADA concentrations. **e** ADA assay signal (counts) using a ECL-based ligand binding assay

To understand the ability of the LC-MS method to detect and isotype actual ADA responses, nonclinical samples from monkeys administered REGN-P or REGN-N were assessed in the LC-MS assay. In one study, monkeys were administered REGN-P intravenously every week for 26 weeks. Two animals that had positive ADA responses detected by an ECL-based ligand binding assay were chosen to be evaluated by our LC-MS method. For monkey 1, a strong positive response was detected in the LBA assay, whereas a weakly positive response was detected in monkey 2 (Fig. 6e). In terms of the drug concentration-time profile for monkey 1, there was an accelerated decrease in serum concentrations which likely reflects the ADA impact on the circulating drug levels. (Fig. 6a). For this monkey with accelerated drug clearance, ADA isotypes IgG1, IgG2, and IgG4 were detected by the LC-MS method. IgG1 was the most abundant isotype (Fig. 6b). It was detected on study day 43 and generally increased throughout the study. IgG2 and IgG4 showed similar increases from day 43 to day 134. Notably, starting from day 57, the concentration of REGN-P in monkey 1 serum samples was below the assay drug tolerance limit of 195 ng/mL (Table II).

**Table II:** Summary of REGN-P Drug Levels, Mass Spec Peak Areas of All ADA Isotypes, Converted ADA Concentrations, and Signal-to-Noise Ratios

Days		0	43	57	71	85	106	120	134	148	162
Monkey 1	Drug level (μg/mL)	BLQ	1.94	0.174	BLQ	BLQ	BLQ	BLQ	BLQ	BLQ	BLQ
	Peak area	278	3309	10,217	20,911	28,106	33,451	39,392	42,783	54,189	55,203
	ADA conc. (μg/mL)	0.03	0.34	1.06	2.17	2.92	3.48	4.10	4.45	5.64	5.75
	Signal/noise	1.0	11.9	36.8	75.2	101.1	120.3	141.7	153.9	194.9	198.6
Monkey 2	Drug level (μg/mL)	BLQ	20.6	24.6	23.4	20.2	10	4.79	0.828	0.396	0.124
	Peak area	829	1231	2002	1160	1521	3425	3371	4443	5507	6986
	ADA conc. (μg/mL)	0.08	0.13	0.21	0.12	0.16	0.35	0.35	0.46	0.57	0.72
	Signal/noise	1.0	1.5	2.4	1.4	1.8	4.1	4.1	5.4	6.6	8.4

*The signal/noise represents the ratio of peak area of a given sample to the pre-dose negative monkey serum sample*

For monkey 2, relatively little ADAs were detected with the LC-MS method which is consistent with the low responses in the LBA assay and the lack of accelerated clearance observed in the concentration vs time profile. Nevertheless, as with monkey 1, IgG1 was the most abundant isotype, followed by IgG4 and IgG2 (Fig. 6c). It should be noted that the REGN-P drug concentrations for monkey 2 were above the assay drug tolerance limit of 195 ng/mL for the majority of the analyzed time points, and therefore, the levels of ADAs in these time points may be underestimated. (Table II).

After successfully isotyping ADAs, the peak areas were summed for all isotypes to calculate the total ADA concentrations in the REGN-P-dosed monkey serum samples using the REGN-P-captured ADA-PC calibration curve (Fig. 6d). Pre-dose serum samples were used as individual negative controls. A signal-to-noise ratio was calculated by comparing sample peak areas to the negative control peak area. Serum samples from monkey 1 were strongly ADA-positive starting from day 43, with blood REGN-P levels at 1.94 μg/mL (Table II). Total ADA concentrations ranged from 340 ng/mL at day 43 to 5.75 μg/mL on day 162. Monkey 2 serum samples were weakly ADA-positive starting on day 106, with blood REGN-P levels at 10 μg/mL. The total ADA concentrations ranged from 350 ng/mL at day 106 to 720 ng/mL on day 162. Monkey 1 had a rapid increase in the levels of ADAs throughout the dosing period, corresponding to its steep decline in drug concentrations early on, whereas monkey 2 has very low levels of ADAs over the same time frame (Table II). Thus, the results obtained using the LC-MS based ADA quantitation method for both monkey 1 and monkey 2 were similar to those obtained with the ligand binding assay (Fig. 6e).

**Fig. 7:**
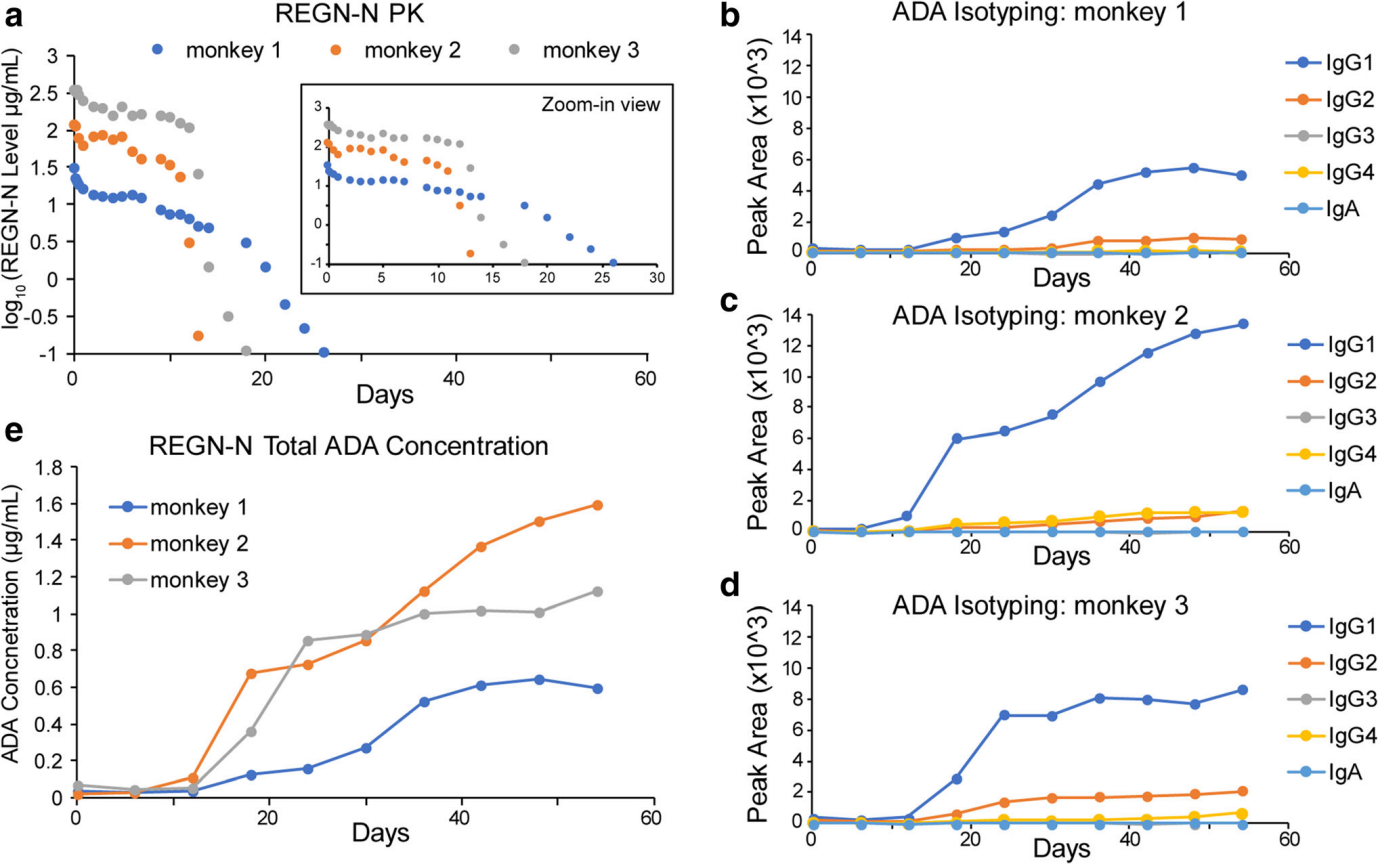
REGN-N monkey serum sample ADA isotyping and quantitation. **a** PK results of monkeys 1, 2, and 3 with a single-dose of 1, 5, and 15 mg/kg REGN-N, respectively. ADA isotyping results using the immunocapture LC-MS method for monkey 1 **b**, monkey 2 **c**, and monkey 3 **d**. **e** ADAs were quantified using the calibration curve in Fig. 4b to convert peak areas to concentrations

Nonclinical serum samples from animals administered REGN-N were also evaluated. Samples from three monkeys collected after a single-dose of REGN-N at doses of 1, 5, or 15 mg/kg were analyzed. All three monkeys demonstrated a rapid decrease in REGN-N drug concentrations over time (Fig. 7a) and all animals exhibited robust positive responses in the LBA ADA assay (not shown). The LC-MS ADA method also detected strong ADA responses in all 3 animals. The isotyping results suggested IgG1 was the most abundant ADA isotype for all these monkeys, with IgG2 presented in all three samples (Fig. 7b, c & d). IgG4 was at a comparable level to IgG2 for monkey 2 but was significantly lower for monkey 3. All three monkeys were ADA-positive beginning on day 18, with ADA concentrations increasing over time (Fig. 7e). It should be noted that the REGN-N drug concentrations in the three monkeys were all below the drug tolerance limit (3.1 μg/mL) for ADA detection.

## DISCUSSION

The optimization of the ADA immunocapture procedure with maximal recovery and minimal matrix interference is critical for the LC-MS ADA assay. Our results suggested direct cross-linking conjugation of drugs onto magnetic beads increased immunocapture recovery rate by twofold compared to the traditional conjugation of biotinylated drugs onto streptavidin magnetic beads (Fig. 2). This is most likely due to poorer labeling efficacy of biotin to drugs compared to cross-linking and due to higher conjugation density of cross-linking compared to bulkier biotin tags. The percentage of unlabeled drugs in both biotinylated REGN-P and REGN-N reagents was approximately 5% (data not shown). The unbiotinylated drugs in the capture reagents would reduce the ADA capture recovery rate as the unbiotinylated drugs could not bind to streptavidin beads and thus were washed away along with any ADAs that bind to the unbiotinylated drugs. In contrast, uncross-linked drugs were washed away from the beads prior to the immunocapture step, resulting in a higher and more consistent ADA capture recovery rate.

Interestingly, when the full-length mAb drug was used as the capture reagent, there was a high level of IgG4 background noise. However, using the Fab fragment in the immunocapture step substantially reduced the background signal (Fig. 3). Human IgG4 can form Fc-Fc contacts which can cause interference in immunoassays, including ADA assays (27–29). The arginine at position 409 (EU numbering) is responsible for these Fc-Fc contacts, and the monkey IgG4 sequence is conserved with the human sequence at this residue (30). The results presented here indicate that Fc-Fc interactions also occur between monkey and human IgG4, but not with human IgG1.

Interference from endogenous monkey IgM was also observed in the immunocapture step. Our results suggest endogenous IgM binds to both IgG1 and IgG4 drugs and acid dissociation was unsuccessful in eliminating these interactions. When using the Fab-only drug as a capture reagent, interference levels increased twofold. Binding to the full-length mAb in the immunocapture step may be due to endogenous anti-human IgM in monkey serum, or RF-type responses directed to the Fc, as occur in humans (31). The increased binding to the Fab version of the drug may have been due to pre-existing anti-hinge antibodies that are not detected with the full-length mAb (32). We were not able to select surrogate peptides for IgE due to the lack of commercially available cynomolgus monkey IgE.

The assay can be used to determine whether a sample is ADA-positive or negative by comparing assay response of a post-dose sample to that monkey’s baseline response, which is sometimes used in nonclinical ADA assessment with LBA assays. For example, we can establish a threshold to determine ADA positivity if the ratio of the MS signals of post-dose samples to the MS signal of the pre-dose samples of the same animal is greater than 3. Using this threshold, REGN-P-dosed monkey 1 had a positive ADA response before day 43, while the first ADA-positive response for monkey 2 was observed at day 106 (Table II).

The assay can tolerate 195 ng/mL of REGN-P and REGN-N in monkey serum without affecting ADA quantitation and can tolerate 3.1 μg/mL of REGN-P and REGN-N in monkey serum without affecting ADA identification (Fig. 5). To further improve the drug tolerance and thus enable ADA quantitation at high drug concentrations, the sample could be pre-treated with anti-human antibodies to deplete the existing drugs. However, the capturing anti-human antibodies should not compete with endogenous ADAs for the same binding domain on the drug. Furthermore, a calibration curve was successfully used to quantify ADA levels with a dynamic range from 123 ng/mL (REGN-P) or 41 ng/mL (REGN-N) to 10 μg/mL. We speculate that stable heavy isotope-labeled ADA-PC can be spiked into serum samples to further improve the quantitation. Furthermore, since ADA-PC technically only represents IgG1 isotype, it will be ideal to generate a positive control cocktail composed of multiple ADA isotype positive standards against human IgG to achieve better quantitation.

## CONCLUSION

An immunocapture LC-MS-based method was successfully employed to isotype and quantify ADAs in monkey serum samples that were previously determined to be ADA-positive by a ligand binding assay. Consistent with previous work (9), our data suggest IgG1 is the predominant ADA isotype. IgG2 and IgG4 were also detected at lower levels, while IgG3 and IgA levels were negligible.

A monkey IgG1 mAb against human kappa light chain was used as a positive control for assay development and as a calibration standard. Several critical experimental conditions in the immunocapture step were optimized. Cross-linking conjugation of drugs to beads ensured maximal ADA recovery. A Fab-only drug was used as the capture reagent to eliminate the interference due to Fc interactions with IgG4 drugs. The multiplex LC-MRM-MS method allows simultaneously to monitor 6 ADA isotypes using 17 MRM transitions with a short 15-min LC method.

In summary, this immunocapture LC-MS assay can provide specific ADA isotype and semi-quantitation information and can be used as a supplementary and orthogonal approach to traditional ADA assays for immunogenicity assessment.

## Supplementary Information

ESM 1 (DOCX 123 kb)
