# Supplementary material for: Isotyping and Semi-Quantitation of Monkey Anti-Drug Antibodies by Immunocapture Liquid Chromatography-Mass Spectrometry
Source: AAPS J. 2021 Jan 6;23(1):16. doi: 10.1208/s12248-020-00538-w (PMC7788027; doi:10.1208/s12248-020-00538-w)
Supplement: Supplementary file 1 — (DOCX 123 kb) [file 12248_2020_538_MOESM1_ESM.docx]

**Figure S1: IgM MRM Transition Development Workflow**

**
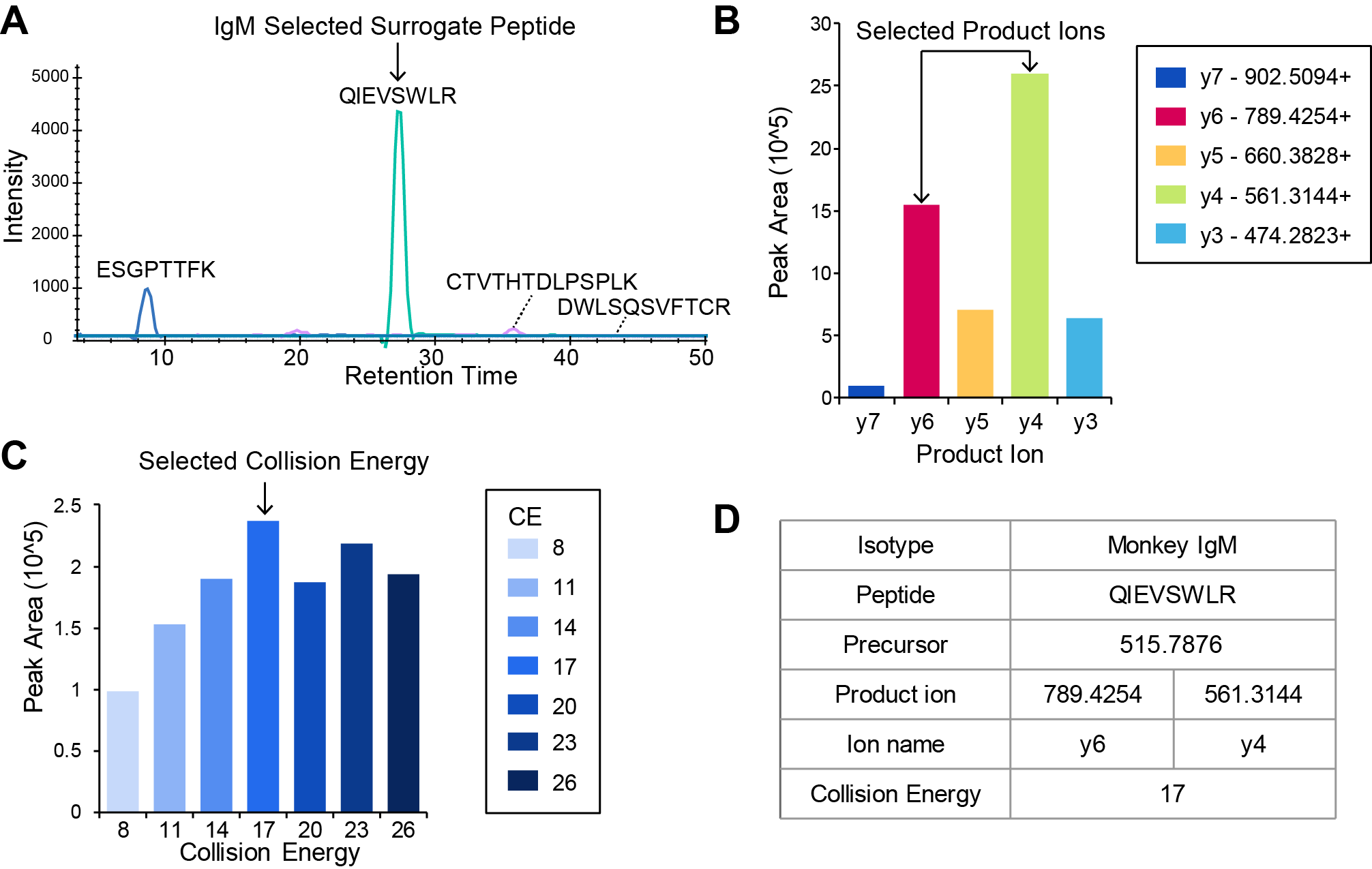
**

(A) Comparing the four IgM unique peptide intensities, QIEVSWLR was selected as the surrogate peptide. (B) Comparing five product ions after fragmentation of QIEVSWLR, the top 2 ions with the highest intensities, y6 and y4, were chosen to generate the MRM transitions. (C) The collision energy that generated the highest intense product ion was used for the MRM method. (D) A summary of optimized MRM transitions for monkey IgM isotype.

**Table S1: List of Cynomolgus Monkey Confirming Peptides, MRM Transitions and Optimized Collision Energies that are also monitored in the Immunocapture-LC-MS Assay**

| **Isotype** | **Peptide** | **Precursor** | **Product Ion** | **Collision Energy** | **Note** |
| --- | --- | --- | --- | --- | --- |
| IgG | STSESTAALGCLVK | 475.2414 | 689.4015 | 12.3 | Common for IgG1, IgG3 and IgG4; Confirming peptide |
|  |  |  | 576.3174 | 12.3 |  |
|  | VVSVLTVTHQDWLNGK | 599.3282 | 617.3406 | 25.8 | Common for all IgGs; Confirming peptide |
|  |  |  | 318.1772 | 25.8 |  |
| IgM | DWLSQSVFTCR | 699.8272 | 984.4567 | 25.7 | Unique for IgM; Confirming peptide |
|  |  |  | 583.2657 | 25.7 |  |
|  | CTVTHTDLPSPLK | 490.2537 | 541.3344 | 15.8 | Unique for IgM; Confirming peptide |
|  |  |  | 147.1128 | 15.8 |  |
| IgA | GFSPEDVLVR | 559.7957 | 827.4621 | 12.4 | Unique for IgA; Confirming peptide |
|  |  |  | 414.2347 | 12.4 |  |
